# *In vivo* functional analysis of L-rhamnose metabolic pathway in *Aspergillus niger*: a tool to identify the potential inducer of RhaR

**DOI:** 10.1186/s12866-017-1118-z

**Published:** 2017-11-06

**Authors:** Claire Khosravi, Roland Sándor Kun, Jaap Visser, María Victoria Aguilar-Pontes, Ronald P. de Vries, Evy Battaglia

**Affiliations:** Fungal Physiology, Westerdijk Fungal Biodiversity Institute & Fungal Molecular Physiology, Utrecht University, Uppsalalaan 8, 3584 CT Utrecht, The Netherlands

**Keywords:** L- rhamnose catabolic pathway, Inducer, Transcriptomics, Pectinase, Gene regulation

## Abstract

**Background:**

The genes of the non-phosphorylative L-rhamnose catabolic pathway have been identified for several yeast species. In *Schefferomyces stipitis*, all L-rhamnose pathway genes are organized in a cluster, which is conserved in *Aspergillus niger*, except for the *lra-4* ortholog (*lraD*). The *A. niger* cluster also contains the gene encoding the L-rhamnose responsive transcription factor (RhaR) that has been shown to control the expression of genes involved in L-rhamnose release and catabolism.

**Result:**

In this paper, we confirmed the function of the first three putative L-rhamnose utilisation genes from *A. niger* through gene deletion. We explored the identity of the inducer of the pathway regulator (RhaR) through expression analysis of the deletion mutants grown in transfer experiments to L-rhamnose and L-rhamnonate. Reduced expression of L-rhamnose-induced genes on L-rhamnose in *lraA* and *lraB* deletion strains, but not on L-rhamnonate (the product of LraB), demonstrate that the inducer of the pathway is of L-rhamnonate or a compound downstream of it. Reduced expression of these genes in the *lraC* deletion strain on L-rhamnonate show that it is in fact a downstream product of L-rhamnonate.

**Conclusion:**

This work showed that the inducer of RhaR is beyond L-rhamnonate dehydratase (LraC) and is likely to be the 2-keto-3-L-deoxyrhamnonate.

**Electronic supplementary material:**

The online version of this article (doi: 10.1186/s12866-017-1118-z) contains supplementary material, which is available to authorized users.

## Background

Plant biomass is mainly composed of polysaccharides. In nature, fungi secrete a broad range of polysaccharide degrading enzymes to release monosaccharides, which are then used as nutrients. Pectin is a complex plant cell wall polysaccharide that can be divided into four sub-structures: homogalacturonan (HGA), xylogalacturonan (XGA) and rhamnogalacturonan I and II (RG-I and RG-II) [1]. The backbone of RG-I is composed of alternating L-rhamnose and D-galacturonic acid residues. Long side chains of L-arabinose (arabinan), D-galactose (galactan), or a mixture of L-arabinose and D-galactose residues (arabinogalactan) can be attached to these L-rhamnose residues [2, 3].

*A. niger* is able to efficiently degrade pectin and can utilize L-rhamnose also as the sole carbon source. *A. niger* possesses enzymes that are able to enzymatically release L-rhamnose from RG-I. Endo- and exo-rhamnogalacturonase, α-rhamnosidase, rhamnogalacturonan lyase and rhamnogalacturonan acetyl esterase are all active towards the main chain of RG-I [4, 5].

An L-rhamnose transporter (RhtA) has been characterized recently in *A. niger* [6]. Interestingly, it has been found that *rhtA* is co-localized with the α-L-rhamnosidase gene *rhaB*. The transcriptional profile of *rhtA* and *rhaB* genes during a time-course growth experiment of *A. niger* on L-rhamnose suggests not only that there is a coordinated role in the release and the transport of L-rhamnose during consumption but also that strong activation of these two genes do not require high concentrations of L-rhamnose but only low levels [6].

**Fig. 1:**
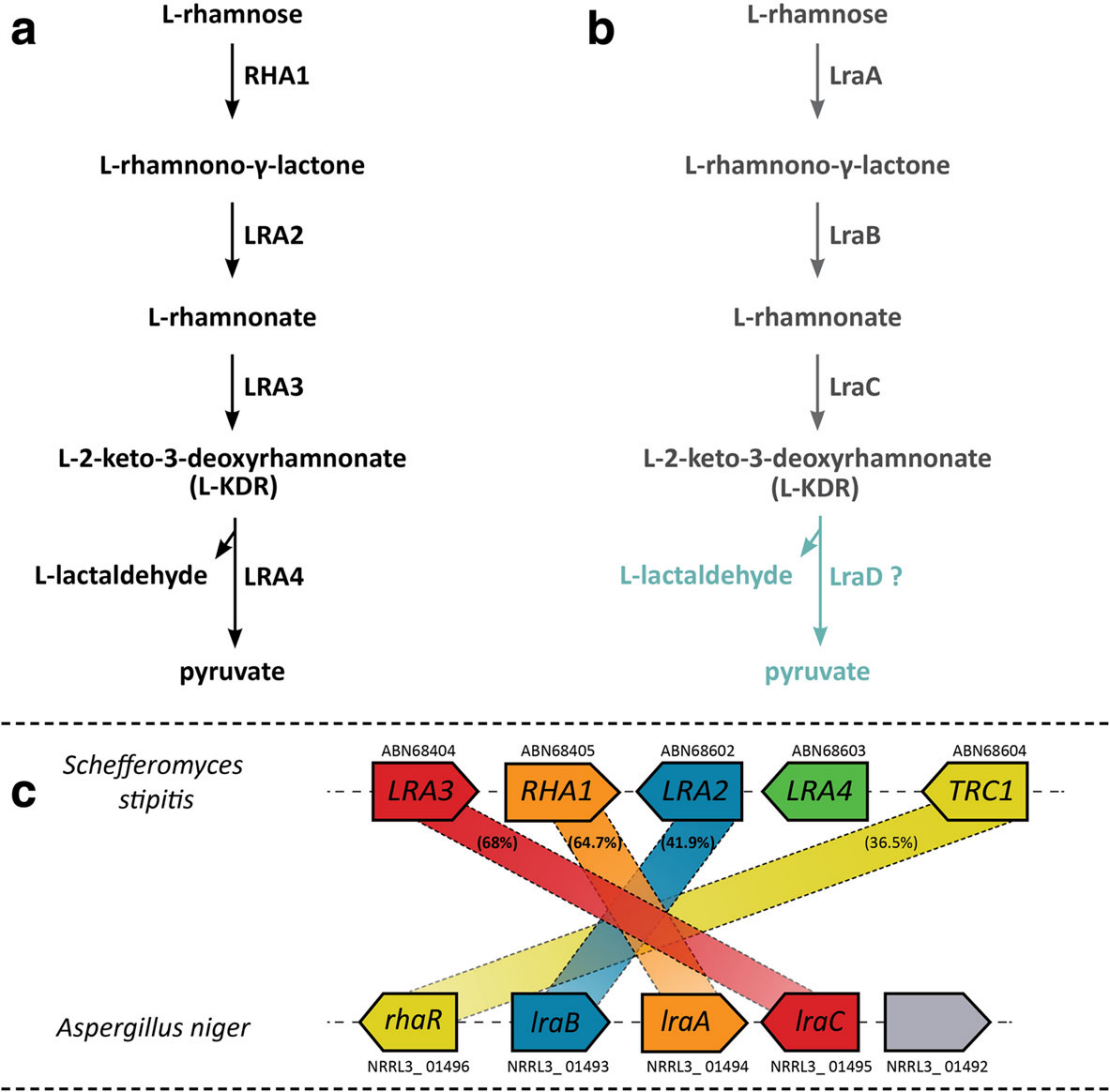
Schematic representation of the L-rhamnose metabolic pathway in *Schefferomyces Stipitis* (**a**) and in *Aspergillus niger* (**b**). Orientation of the genes in the gene cluster in *S.stipitis* and *A. niger* (**c**) (LraA = L-rhamnose-1-dehydrogenase, LraB = L-rhamnono-γ-lactonase, LraC = L-rhamnonate dehydratase, LraD = L-2-keto-3-deoxyrhamnonate aldolase (L-KDR aldolase).

Two pathways are known for the catabolism of L-rhamnose: a phosphorylative and a non-phosphorylative pathway. The phosphorylative pathway is only found in bacteria such as *Escherichia coli* [7], while the non-phosphorylative pathway is found in bacteria and yeasts [8–12]. In the non-phosphorylative pathway of *Schefferomyces stipitis*, L-rhamnose is converted to pyruvate and L-lactaldehyde via four metabolic reactions involving a L-rhamnose-1-dehydrogenase (RHA1) that oxidizes L-rhamnose to L-rhamnono-Y-lactone, a L-rhamnono-γ-lactonase (LRA2) that converts L-rhamnono-γ-lactone to L-rhamnonate, a L-rhamnonate dehydratase (LRA3) that converts L-rhamnonate to 2-keto-3-deoxy-L-rhamnonate (L-KDR) and a 2-keto-3-deoxy-L-rhamnonate (L-KDR) aldolase (LRA4) that converts the 2-keto-3-deoxy intermediate to pyruvate and L-lactaldehyde (Fig. 1a). All the genes (RHA1, LRA2, LRA3 and LRA4) have been identified in *S. stipitis* and the corresponding enzymes have been biochemically characterized [8, 13, 14]. The RHA1, LRA2, LRA3, LRA4 genes are organized in a cluster in *S. stipitis* together with a transcription factor with a Zn (II)2Cys6 zinc binuclear cluster domain TRC1 (Fig. 1c). This gene cluster is either fully or partially characterized in other fungal species [13]. In *A. niger*, the homologous genes of RHA1, LRA2, LRA3 designated as *lraA*, *lraB* and *lraC,* as well as the L-rhamnose regulator RhaR [15] are organized in a cluster (Fig. 1c). However, no LRA4 homolog has been found in the cluster in the *A. niger* genome (Fig. 1b). Only one of the *A. niger* L-rhamnose metabolic genes, *lraC*, has been biochemically characterized to encode a L-rhamnonate dehydratase by expressing it in *Saccharomyces cerevisiae* [16]. A double deletion of *lraC* and *lraA* in *A. niger* resulted in a strain that exhibited no L-rhamnose dehydrogenase activity and could not consume or grow on L-rhamnose [17]. It remains unknown which gene function, *lraA* or *lraC*, is actually the cause of the inability of *A. niger* to grow on L-rhamnose. As the in vivo function of none of the three individual L-rhamnose pathway genes *(lraA*, *lraB* and *lraC*) has been studied yet in *A. niger* our first goal was to perform an in vivo analysis of their function by making single gene deletion mutants in *A. niger*. Secondly, we searched and selected *lraD* candidate genes in the *A. niger* genome for making the corresponding gene deletions. And thirdly we made use of the metabolic mutants to identify the inducer of RhaR in *A. niger*. In *A. niger*, RhaR has been shown to control expression of genes involved in RG-I degradation, the L-rhamnose transporter gene (*rhtA*) as well as the L-rhamnose catabolic genes (*lraA*, *lraB* and *lraC*) during growth on L-rhamnose [6, 15, 18]. It has been suggested that L-rhamnose or a conversion product thereof could be the inducer of RhaR [15]. Blocking the individual steps in the L-rhamnose pathway would thus allow the identification of the inducer. Either the sugar itself or a metabolic pathway intermediate has been shown to induce other plant biomass related transcriptional regulators, such as L-arabinose/L-arabitol for AraR [19], D-xylose for XlnR [20] and 2-keto-3-deoxy-L-galactonate for GaaR-GaaX activator-repressor module [21]. Therefore, we did not make the deletion mutant of *rhtA* or any other putative L-rhamnose transporter in order to allow always L-rhamnose uptake.

Gene deletion and transcriptomic analysis performed in this study showed that *lraA*, *lraB* and *lraC* are required for growth on L-rhamnose and confirmed that RhaR plays an important role in the regulation of L-rhamnose catabolism and the degradation of pectin. The results also indicate that L-rhamnose, L-rhamnono-Y-lactone and L-rhamnonate are not the inducers of RhaR, but that this is a compound located further down in the metabolic pathway.

## Methods

### Strains, media and culture conditions

*A. niger* strain CBS 141257 (Table S1) was used to create the Δ*lraA*, Δ*lraB* and Δ*lraC* strains. The CBS 141257 strain was obtained by transformation of N593.20 with uridine (*pyrG)* from *Aspergillus oryzae* [22]. All *A. niger* strains were grown at 30 °C using minimal medium (MM, pH 6) or complete medium (CM, pH 6) [23] with 1.5% of agar. Spores plates contained CM with 2% D-glucose. Plates used in the growth profile contained MM + 25 mM monosaccharides, inoculated with 1000 spores.

Liquid cultures of two biological duplicates were inoculated with 10^6^spores/ml and incubated in a rotary shaker at 250 rpm and 30 °C. Pre-cultures for RNA isolation were grown for 16 h in 1 L Erlenmeyer flasks containing 250 ml CM with 2% D-fructose. Mycelium was washed with MM and transferred for 2 h in 250 ml Erlenmeyer flasks containing 50 ml MM supplemented with 25 mM L-rhamnose for RNA-seq. For qPCR analysis, mycelium was transferred for 2 h in 50 ml Erlenmeyer flaks containing 10 ml MM supplemented with 25 mM carbon source L-rhamnose (Fluka) or L-rhamnonate (Sigma). Mycelium was harvested by vacuum filtration, dried between tissue paper and frozen in liquid nitrogen. New strains were deposited at the Westerdijk Fungal Biodiversity Institute with strain numbers indicated in Additional file 1: Table S1.

### Molecular biology methods and fungal transformation

To construct the deletion cassettes, the upstream and downstream flanks of the genes (*lraA*, *lraB* and *lraC*) were amplified using PCR with gene specific primers (Additional file 2: Table S2). The upstream flank reverse primer and downstream flank forward primer carried homologous sequences, overlapping the ends of the *pyrG* selection cassette. The *pyrG* cassette was amplified from pRV1005 and purified using the Wizard_ SV Gel and PCR Clean-Up Start-Up Kits (Promega). These three fragments were combined in a fusion PCR reaction, to generate the deletion cassette. The fusion PCR mixture contained 1 μl of each PCR product, 12.5 μL GoTaq_ Long PCR Master Mix (Promega), 2 μL of each 10 mM primer in a total volume of 25 μL. The following PCR conditions were used: 2 min at 94 °C, 20 s at 94 °C, 20 s at 58 °C, 5 min at 68 °C for 30 cycles and finally an additional 10 min at 68 °C. The amplified deletion cassettes were purified using the Wizard_ SV Gel and PCR Clean-Up Start-Up Kits (Promega). Purified DNA (5 μg) of the deletion cassette was used to transform *A. niger*. Protoplast-mediated transformations of *A. niger* were performed as described [24]. DNA was isolated from frozen mycelium, ground with a Tissue Lyser (QIAGEN) using a standard chloroform/phenol extraction. For screening of the *A. niger* transformants, a PCR was performed using genomic DNA of the transformants. Gene-specific sets of primers were used to check the absence or presence of the ORF (Additional file 2: Table S2). The resulting strains are listed in Additional file 1: Table S1.

### RNA extraction, cDNA library preparation and RNA-seq

Total RNA was extracted from mycelium ground in a Tissue Lyser (QIAGEN) using the TRIzol reagent (Invitrogen, Breda, The Netherlands) according to the instructions of the manufacturer. Total RNA samples were purified with the NucleoSpin RNA Clean-up Kit (Macherey-Nagel). Contaminating gDNA was removed by an rDNase solution directly on the silica membrane. RNA integrity and quantity were analyzed on a 1% agarose gel using gel electrophoresis and with the RNA6000 Nano Assay using the Agilent 2100 Bioanalyzer (Agilent Technologies). cDNA library preparation and sequencing reactions were conducted at BGI Tech Solutions Co., Ltd. (Hong Kong). Illumina library preparation, clustering, and sequencing reagents were used throughout the process following the manufacturer’s recommendations (http://illumina.com). mRNA was purified using poly-T oligonucleotide-attached magnetic beads and then fragmented. The first and second strands cDNA were synthesized and end repaired. Adaptors were ligated after adenylation at the 3′ end. After gel purification, cDNA templates were enriched by PCR. cDNA libraries were validated using the Agilent 2100 Bioanalyzer (Agilent Technologies) and quantified by qPCR. Single-read samples were sequenced using Illumina HiSeq™ 2000 platform (http://illumina.com). On average 51 bp sequenced reads were constituted, producing approximately a yield of 360 MB raw sequence for each sample.

### RNA-seq data analysis and functional annotation

Raw reads were produced from the original image data by base calling. After data filtering, the adaptor sequences, highly ‘N’ containing reads (> 10% of unknown bases) and low quality reads (more than 50% bases with quality value of < 5%) were removed. After data filtering, on average, ∼ 99% clean reads remained in each sample. Clean reads were then mapped to the genome of *A. niger* NRRL3 (http://genome.jgi.doe.gov/Aspni_NRRL3_1/Aspni_NRRL3_1.home.html) using BWA [25, 26]. No more than two mismatches were allowed in the alignment. On average, 70% total mapped reads to the gene was achieved. We refer to *A. niger* gene IDs based on the most up-to-date and accurate annotation of the *A. niger* NRRL3 genome (http://genome.fungalgenomics.ca/). The gene expression level (FPKM) was calculated by using RSEM tool [27]. Genes with expression value higher than 120 were considered highly expressed (approximately top 5%) and differential expression was identified by Student’s t-test with a *P*-value cutoff 0.05. The RNA-seq data have been submitted to Gene Expression Omnibus (GEO) [28] with accession number: GSE99865.

### qRT-PCR analysis

cDNA was prepared from total RNA (2.5 μg) using Thermoscript RT (Invitrogen) according to the instructions of the manufacturer. The sequences of all primers for qRT-PCR analysis were designed using the Primer Express 3.0 software (Applied Biosystems). The primers were tested to determine the optimal primer concentrations and efficiency. Combinations of the 50 nM, 300 nM and 900 nM (final concentration) per primer pair were checked, and based on the dissociation curve the optimal primer concentration per primer pair was chosen. The primer sequences of the tested genes and the reference gene are listed in Additional file 3: Table S3. qPCR analysis was performed by using the ABI 7500 fast real-time PCR system (Applied Biosystems). The reactions consisted of 2 μl forward and reverse primers at optimal concentration, 20 ng cDNA sample, 10 μl ABI Fast SYBR Master Mix (Applied Biosystems), and water to a final volume of 20 μl. The cycling parameters were 95 °C for 20 s, followed by 40 cycles of 95 °C for 3 s and 60 °C for 30 s. A dissociation curve was generated to verify that a single product was amplified. Transcript levels were normalized against the histone H2B gene expression and quantified according to the formula 2 –(Ct gene X – Ct H2B) [29]. Two biological and three technical replicates were analyzed.

## Results

### In vivo effects of deleting the L-rhamnose metabolic genes

**Fig. 2:**
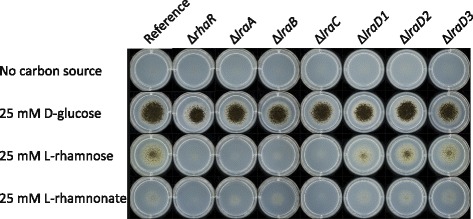
Growth profile on L-rhamnose, L-rhamnonate and D-glucose of the reference strain and the deletion mutants. The reference, Δ*lraA*, Δ*lraD1*, Δ*lraD2* and Δ*lraD3* were grown on MM with 25 mM L-rhamnose, 25 mM L-rhamnonate and 25 mM D-glucose for 4 days at 30 °C. Spore inoculations were done with 1000 spores

The candidate genes for the first three enzymatic reactions of the L-rhamnose pathway are homologs of the characterized genes of *S. stipitis* [15]. The single deletions in NRRL3_08837 (Δ*lraA*), NRRL3_01493 (Δ*lraB)* and NRRL3_10522 (Δ*lraC)* resulted in strains that are unable to grow on L-rhamnose (Fig. 2) compared to the reference strain. A similar growth phenotype was observed on L-rhamnose for the Δ*rhaR* strain (Fig. 2). The small colonies observed for the metabolic deletion mutants on L-rhamnose is background growth since the plates looked similar to our control agar plates without any carbon source added (Fig. 2). Growth of the metabolic deletion mutants on D-glucose was similar to the reference (Fig. 2). This result indicates that all three genes of the pathway are essential for the conversion of L-rhamnose. Growth of Δ*lraA* and Δ*lraB* was reduced on L-rhamnonate, while no growth was observed in the Δ*rhaR* and Δ*lraC* on L-rhamnonate (Fig. 2).

Previously, it was already shown by performing a bidirectional BLAST analysis that the closest homolog of *S. stipitis* LRA4 is NRRL_08779 [15]. However, this gene is not found in the *A. niger* L-rhamnose cluster (Fig. 1c), it is not induced on L-rhamnose compared to D-glucose and its expression is not affected in the Δ*rhaR* mutant on L-rhamnose compared to the reference strain (Additional file 4: Table S4). In addition, it has a very low expression level in all growth conditions tested. In order to search for candidate genes for the L-KDR aldolase (*lraD*), we analyzed the *A. niger* genome for genes containing any of the InterPro and PFAM domains (Additional file 4: Table S4), similar domains to those found in *S. stipitis* LRA4 [13]. The most similar gene to *S. stipitis* LRA4, NRRL_08779, is not significantly expressed on L-rhamnose in the micro-array data, nor in any other in-house micro-array or RNAseq datasets, and was also not detected by qPCR on either L-rhamnose or L-rhamnonate (data not shown), and was therefore excluded as a candidate for this enzymatic function in *A. niger*. We therefore compared the expression between L-rhamnose and D-glucose of all candidate genes containing the relevant domains and ranked them by fold-change between L-rhamnose and D-glucose, resulting in the selection of three candidate genes (NRRL3_03899, NRRL3_05649, NRRL3_06731). The first candidate was 39-fold up-regulated, the second one was 24-fold up-regulated and the third one was 3-fold up regulated on L-rhamnose in comparison to D-glucose (Additional file 4: Table S4). The other genes were either not induced on L-rhamnose compared to D-glucose or not expressed at all. These three candidate genes for *lraD* were deleted in the *A. niger* CBS 141257. Growth phenotypic analysis of the mutants (Δ*lraD1*, Δ*lraD2,* Δ*lraD3*) was performed and growth on L-rhamnose was compared to the reference strain. Deletion of the genes did not affect growth on L-rhamnose and L-rhamnonate (Fig. 2), suggesting that these genes are not the functional homologs of *S. stipitis* LRA4.

### L-KDR is most likely the inducer of the pathway specific regulator RhaR

**Fig. 3:**
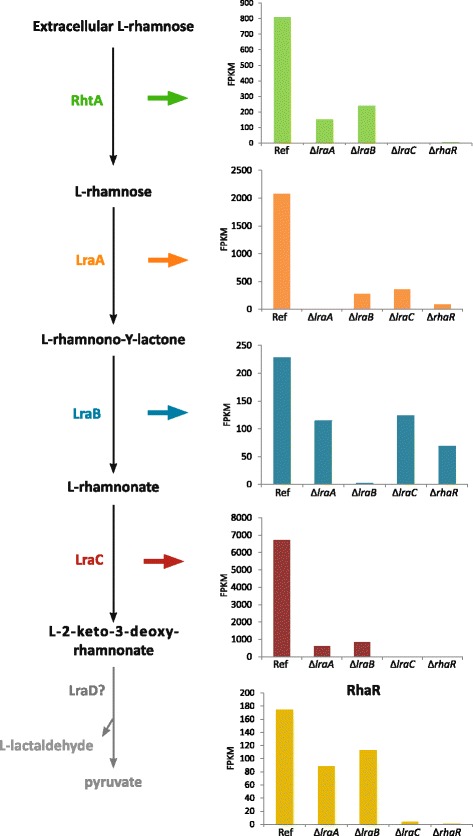
Schematic representation of the expression of *rhtA*, *lraA, lraB* and *lraC* in the L-rhamnose pathway and *rhaR* in *A. niger* Δ*lraA*, Δ*lraB*, Δ*lraC* and Δ*rhaR* strains after 2 h of transfer from fructose to liquid medium containing 25 mM of L-rhamnose*.* Gene expression values are presented in FPKM

RhaR was previously identified as an L-rhamnose-responsive transcription factor gene with a role in induction of genes involved in L-rhamnose transport, catabolism and in RG-I degradation [6, 15]. To test the effect of the three metabolic gene deletions on the induction of L-rhamnose responsive genes, RNA-seq analysis was performed. The reference strain and the KO strains Δ*lraA*, Δ*lraB*, Δ*lraC* and Δ*rhaR* were pre-grown in liquid cultures containing MM with fructose and then transferred to MM with L-rhamnose. RNA-seq analysis showed that the expression levels of *lraA*, *lraB* and *lraC* were close to 0, in Δ*lraA*, Δ*lraB* and Δ*lraC*, respectively (Fig. 3) confirming the deletion of these genes. When *lraA, lraB* or *lraC* are deleted*,* the expression level of the other genes of the L-rhamnose pathway is reduced (Fig. 3). A high expression level was observed for *rhaR* in the wild type, which was reduced to almost zero in Δ*lraC*, but only a small reduction in its expression was visible in Δ*lraA* and Δ*lraB* (Fig. 3). In the Δ*rhaR* mutant, expression of *lraA*, *lraB* and *lraC* is strongly reduced, confirming that these genes are under control of RhaR, as previously reported [15]. In the Δ*lraA* and Δ*lraB* mutant, the expression level of the L-rhamnose transporter gene (*rhtA*) is strongly reduced (Fig. 3; Additional file 5: Table S5). However, *rhtA* was not expressed in the Δ*lraC* and Δ*rhaR* mutants (Fig. 3). Only one of the other candidate L-rhamnose transporter genes described previously [6] showed a similar gene expression profile in the reference strain and the metabolic mutants (NRRL3_02828).

**Fig. 4:**
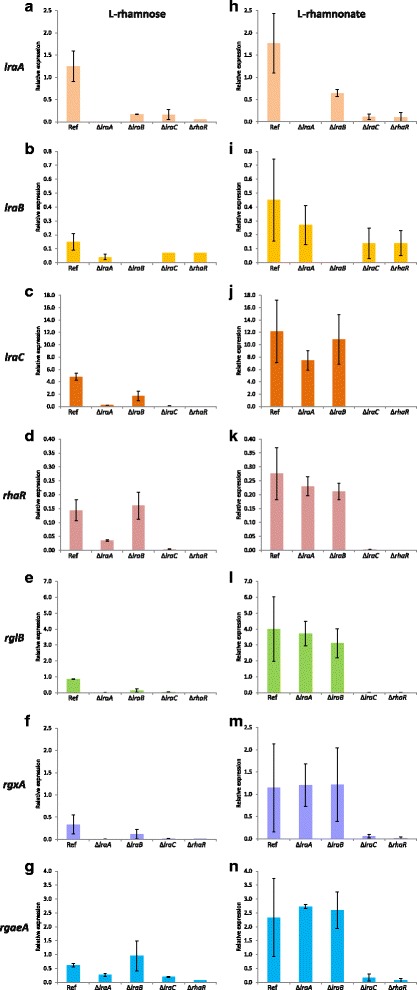
Expression levels of *lraA* (**a**;**h**), *lraB* (**b**;**i**), *lraC* (**c**;**j**), *rhaR* (**d**;**k**), *rglB* (**e**;**l**), *rgxA* (**f**;**m**) and *rgaeA* (**g**;**n**) in *A. niger* Δ*lraA*, Δ*lraB*, Δ*lraC* and Δ*rhaR* mutants *.* The expression was measured in the reference and single mutant strains of *A. niger* after a transfer for 2 h on L-rhamnose (**a-g**) and L-rhamnonate (**h-n**). The columns represent the mean and error bars represent standard deviation between biological replicates

In summary, these results show that in the Δ*lraA*, Δ*lraB* or Δ*lraC* mutant, the transcript level of the other metabolic pathway genes and the transporter gene is reduced. It is most likely that this reduction in gene expression is due to lack of inducer formation and that accumulation of L-rhamnose, L-rhamnono-y-lactone and L-rhamnonate does not result in hyper-induction of RhaR-regulated genes as was seen previously for the *A. niger* Δ*gaaC* mutant during growth on D-galacturonic acid [21]. These results also demonstrate that L-rhamnose, L-rhamnono-γ-lactone and L-rhamnonate are not the inducers of RhaR, and we therefore hypothesized that the inducer is formed further down in the L-rhamnose catabolic pathway and it therefore might be L-KDR. To strengthen this hypothesis, we checked the expression of six rhamnose-induced genes after 2 h of transfer of the metabolic mutants to L-rhamnonate as well. Fructose pre-grown mycelium of the reference, Δ*rhaR*, Δ*lraA*, Δ*lraB* and Δ*lraC* strains were transferred to MM supplemented with either L-rhamnose or L-rhamnonate. qPCR analysis showed reduced expression levels for *lraC*, *lraB*, *lraC*, *rhaR*, rhamnogalacturonan lyase (*rglB)*, exo-rhamnogalacturonan hydrolase *(rgxA)* and rhamnogalacturonan acetylesterase *(rgaeA)* in the *lraA* and *lraC* mutants compared to the reference, after 2 h of culture in L-rhamnose, confirming our RNA-seq data results (Fig. 4a-g). Except for *rhaR* and *rgaeA*, the expression level of the genes is higher in Δ*lraB* compared to the reference strain (Fig. 4d and g). Interestingly, all six genes were induced during 2 h of growth of the reference strain on L-rhamnonate (Fig. 4h-n). Transcript levels were similar to those observed for the reference strain on L-rhamnose (Fig. 4a-g). Deletion of either *lraA* or *lraB* did not affect the expression of the other metabolic genes, the *rhaR* gene and the three RG-I specific genes. However, strongly reduced expression levels of *lraA*, *lraB*, *rhaR*, *rglB*, *rgxA* and *rgaeA* were observed in the Δ*lraC* mutant upon the 2 h transfer to L-rhamnonate compared to the reference strain (Fig. 4h-n).

### CAZy genes involved in degradation of the RG-I were down-regulated in the L-rhamnose pathway mutants

The expression of pectinolytic genes was compared between the L-rhamnose pathway mutants and the reference strain after a transfer for 2 h from fructose to liquid medium containing 25 mM of L-rhamnose to study the transcript profiles of all rhamnose-induced genes in detail. Pectinolytic genes were divided into subclasses, depending on where they act on the pectin backbone: homogalacturonan (HGA), xylogalacturonan (XGA), rhamnogalacturonan-I (RG-I) and side chains (SC) (Additional file 6: Table S6). Most of the genes involved in the degradation of the RG-I backbone were down-regulated in the *lraA*, *lraB*, *lraC* and *rhaR* deletion mutants compared to the reference strain: two genes encoding GH28 exo-rhamnogalacturonases (*rgxA:* NRRL3_02832; *rgxB*: NRRL3_08631), four putative GH78 α-rhamnosidases (NRRL3_02162; NRRL3_06304; NRRL3_03279; NRRL3_07520), one gene encoding a GH105 unsaturated rhamnogalacturonan hydrolase (*urhgA*; NRRL3_00839), one gene encoding a PL4 rhamnogalacturonan lyase (*rglB*; NRRL3_10115) and two genes encoding CE12 rhamnogalacturonan acetyl esterases (*rgaeA*; NRRL3_00169 and *rgaeB*; NRRL3_07501) (Table 1 and Additional file 6: Table S6). Two pectinolytic genes involved in the degradation of HGA and XGA, one PL1 pectin lyase (*pelF*; NRRL3_04153) and one CE12 pectin acetyl esterase (*paeA*; NRRL3_06053), showed a strongly reduced expression level on L-rhamnose in all metabolic deletion strains and in Δ*rhaR* compared to the reference strain (Additional file 6: Table S6). Two genes involved in the degradation of the pectin side chains, one GH3 β-xylosidase gene (*xlnD*; NRRL3_02451) and one GH35 β-1, 4-galactosidase gene (*lacC*; NRRL3_11738) showed a similar transcript profile.

**Table 1:**
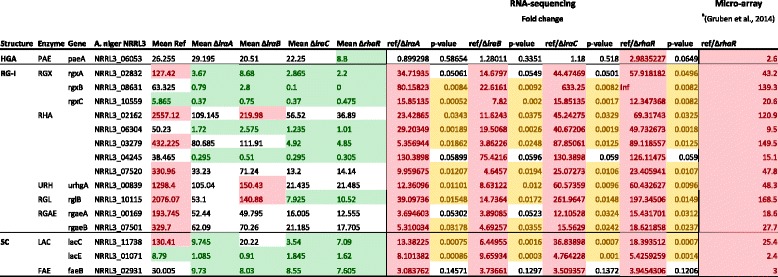
Pectinolytic genes that were significantly down-regulated in the A. niger ΔlraA, ΔlraB, ΔlraC and ΔrhaR strains compared to the reference in L-rhamnose

Based on Gruben, B.S., M. Zhou, A. Wiebenga, J. Ballering, K.M. Overkamp, P.J. Punt & R.P. de Vries, (2014) Aspergillus niger RhaR, a regulator involved in L-rhamnose release and catabolism. Appl Microbiol Biotechnol 98: 5531-5540

The expression levels are mean values of duplicate samples. Genes with values higher than 120 are considered highly expressed and marked red.

Genes with values lower than 20 are considered low expressed and marked green.

The fold change is the difference in expression between the reference strain and the deletion mutants.

The cutoff for differential expression is fold change >1.5 (cells marked red if upregulated and green if downregulated) and p-value <0.05 (cells marked yellow).

HGA homogalacturonan, RG-I Rhamnogalacturonan-I, SC side chains, PAE = pectin acetyl esterase, RGX = exorhamnogalacturonase

RHA = endorhamnogalacturonase, URH = unsaturated rhamnogalacturonyl hydrolase, RGL = rhamnogalacturonan lyase

RGAE = rhamnogalacturonan acetyl esterase, LAC = beta-galactosidase, FAE = feruloyl esterase.

Two GH43 endoarabinases showed different expression profiles, *abnA* (NRRL3_00092) was highly expressed in Δ*lraC* and *abnC* (NRRL3_05407) was up-regulated in Δ*lraA,* Δ*lraB* and Δ*lraC* compared to the reference.

## Discussion

In this study, we constructed single deletion mutants to confirm in vivo the function of the first three genes of the L-rhamnose catabolic pathway in *A. niger* (Δ*lraA*, Δ*lraB* and Δ*lraC*). These strains, together with the Δ*rhaR* mutant, were also used to investigate L-rhamnose-induced transcriptional up-regulation by RhaR in *A. niger* to elucidate the inducer molecule. Comparative growth analysis of the 3 L-rhamnose catabolic mutants showed that individual deletion of *lraA*, *lraB* and *lraC* results in an inability to use L-rhamnose as a sole carbon source. This indicates that there are no other enzymes capable of replacing the function of LraA, LraB and LraC, at least at a level that can rescue the growth phenotype on L-rhamnose. These results are in line with a previous study in which a double deletion mutant of *lraA* and *lraC* in *A. niger* was unable to grow on L-rhamnose [17, 27].

The second aim of our study was to search for *lraD* candidate genes in the *A. niger* genome and identify this function by gene deletion. L-rhamnose catabolism genes (RHA1, LRA2, LRA3 and LRA4) have been previously found in a chromosomal gene cluster in *S. stipitis* [8, 13]. In *A. niger*, the orthologs of the *S. stipitis* RHA1, LRA2 and LRA3 genes, the *lraA, lraB* and *lraC* genes, are clustered with *rhaR* on chromosome II, but the cluster does not contain an LRA4 homolog (*lraD)* [15]. In this study we selected three candidate genes for *lraD* that were specifically up-regulated in L-rhamnose and which all have similar PFAM and InterPro domains to those found in LRA4 of *S. stipitis.* However, deletion of these genes did not reduce growth on L-rhamnose, suggesting that neither of them encodes an L-KDR specific aldolase with a key role in L-rhamnose metabolism. Because the five remaining genes in the *A. niger* genome with a dihydrodipicolinate synthetase family domain (PF00701) were not induced on L-rhamnose or not expressed in any condition (Additional file 4: Table S4), it is very unlikely that these genes are involved in L-rhamnose metabolism in *A. niger*. A possibility is that the real *lraD* gene of *A. niger* belongs to a different aldolase family than the LRA4 of *S. stipitis*. Also, we cannot exclude that an alternative enzyme could convert L-KDR in the L-rhamnose pathway of *A. niger*. In *Sphingomonas sp.*, a gene cluster consisting of LRA1–3, LRA5 and LRA6 has been found. LRA5 and LRA6 were assigned as new enzymes, L-KDR-4-dehydrogenase (KDRDH) and 2, 4-diketo-3-deoxy-L-rhamnonate hydrolase (DDRH), respectively [11]. LRA5 (KDRDH) was identified as an NAD+ specific enzyme belonging to the short-chain dehydrogenase/reductase (SDR) superfamily [11] and has been shown to convert L-KDR to 2, 4-diketo-3-deoxy-L-rhamnonate. Interestingly, another KDRDH belonging to the medium chain dehydrogenase reductase (MDR) superfamily has been biochemically characterized in *Sulfobacillus thermosulfidooxidans* and was found to catalyze the same metabolic reaction [10]. We analysed and compared the PFAM domains in the protein sequences of LRA5 of both species. The KDRDH from *Sphingomonas sp*. contains an enoyl-(Acyl carrier protein) reductase domain (PF13561) and the KDRDH from the acidophile *S. thermosulfidooxidans* contains an alcohol dehydrogenase GroES-like domain (PF08240) and a zinc-binding dehydrogenase domain (PF00107). In *A. niger*, a putative alcohol dehydrogenase (NRRL3_01492) is present in the *lraA-C* cluster (Fig. 1c), which is 33-fold up-regulated in the micro-array data of *A. niger* wild-type in L-rhamnose compared to D-glucose [15]. This putative alcohol dehydrogenase contained the same PFAM domains as KDRDH from *S. thermosulfidooxidans.* Interestingly, this putative alcohol dehydrogenase is well conserved within the genomes of the other Aspergilli [15]. This putative alcohol dehydrogenase might be a likely candidate in *A. niger*, to convert L-KDR into 2, 4-diketo-3-deoxy-L-rhamnonate.

The third aim was to study the transcript profiles of the L-rhamnose-induced genes in the metabolic deletion mutants to identify the inducer of RhaR in *A. niger.* In our RNA-seq analysis, genes involved in the L-rhamnose catabolism and in RG-I degradation were significantly lower expressed in Δ*lraA*, Δ*lraB* and Δ*lraC* mutants compared to the reference strain. Our results revealed that 12 of the 23 RG-I related pectinolytic genes were >1.5 fold down-regulated in all the deletion strains compared to the reference strain on L-rhamnose. In the D-galacturonic acid pathway in *A. niger*, deletion of *gaaA* and *gaaB* resulted in reduced expression profiles of pectinolytic genes on D-galacturonic-acid compared to the reference [21], while deletion of *gaaC* resulted in up-regulation of these genes whereas no difference was observed for the deletion of *gaaD*. This study also demonstrated that the inducer of the galacturonic acid degradation route is 2-keto-3-deoxy-L-galactonate, which is the substrate for GaaC, indicating that accumulation of an intermediate due to deletion of a pathway gene allows the identification of the inducer of the pathway. Since the expression of L-rhamnose-responsive genes was reduced in the strains in which *lraA*, *lraB* or *lraC* were deleted, this indicates that none of these deletions results in accumulation of the inducer of the pathway regulator (RhaR). The expression did not reduce to zero for all the known rhamnose catabolic genes in all the strains, suggesting that there is either a basal non-regulated expression of these genes or alternatively that they are also under control of other regulatory systems. Based on an alignment between TRC1 in *S. stipitis* and RhaR in *A. niger*, regions of the DNA Binding domain are conserved. This correlates with the phylogenetic analysis performed in Koivistoinen et al.,2012. Therefor RhaR appears to be an orthologue of TRC1. Also, since the transcription factor RhaR is conserved within the genomes of the other Aspergilli, and is also conserved in fungal species, we postulate that the product of the LraC reaction, L-KDR, is the inducer of the system. In *S. stipitis* another transcription factor than TRC1 has been found in the L-rhamnose cluster, FST14. This transcription factor was more up-regulated than TRC1 on L-rhamnose and it might be co-regulating the pectinolytic genes together with TRC1 [13]. This could also be the case in *A. niger* as previously suggested [15], even though we could not find an orthologue for this regulator in *A. niger*.

The L-rhamnose transporter encoding gene and the metabolic genes are L-rhamnose-induced and in the qPCR and RNA-seq analysis they have a similar gene expression profile in the metabolic and *rhaR* deletion mutants. These results and those of Sloothaak et al., 2016 [6] indicate that L-rhamnose is predominantly transported via the RhtA transporter and then converted through the L-rhamnose metabolic pathway.

Previously it was shown that only a small concentration of L-rhamnose is enough to induce the system [6], as also observed for the D-xylose regulatory system in *A. niger* [30]. The very low expression levels of *lraA*, *lraB*, *rhaR*, *rglB*, *rgxA*, *rgaeA* obtained in Δ*lraC* in the presence of L-rhamnonate showed that the third step is indeed necessary to generate the inducer. In the Δ*lraA* and Δ*lraB* strains the inability to generate the inducer from L-rhamnose can be overcome by supplying L-rhamnonate instead, which is the product of LraB. This then also explains why Δ*lraA* and Δ*lraB* can still grow on L-rhamnonate, while Δ*lraC* cannot. RNA-seq combined with the qPCR results of the L-rhamnose metabolic mutants demonstrated that L-rhamnose, L-rhamnono-γ-lactone and L-rhamnonate are not the inducers of RhaR. Interestingly and in contrast to transfer to L-rhamnose, upon transfer to L-rhamnonate the reference, Δ*lraA* and Δ*lraB* strains showed similar expression levels in all the genes tested. L-rhamnonate is unlikely to use the L-rhamnose transporter RhtA, due to its different chemical nature. The higher levels of expression observed in the reference, Δ*lraA* and Δ*lraB* strains suggest that by using L-rhamnonate as carbon source which avoids the first 2 steps in the L-rhamnose metabolic pathway, its metabolism still leads to induction of the L-rhamnose pathway. But induction requires a functional LraC since induction is severely affected in the *lraC* deletion strain.

Because the gene or genes involved in the conversion of L-KDR remain unknown in *A. niger*, studying metabolic gene deletion mutants further downstream of the LraC step is required to unambiguously establish the identity of the inducer. At this point in time we do not know whether L-KDR is converted by a yet unknown aldolase into L-lactaldehyde and pyruvate or, alternatively by LRA5 and LRA6 homologs to L-lactate and pyruvate. The putative alcohol dehydrogenase present in the cluster, which on the basis of the PFAM domains found and its rhamnose-responsive expression, makes it more likely that the L-rhamnonate degradation pathway involves formation of 2, 4-diketo-3-deoxy-L-rhamnonate from L-KDR. The final C3 metabolites formed (pyruvate and L-lactaldehyde or L-lactate) via these two non-phoshorylating pathways have to be further metabolized via gluconeogenesis. These metabolites are already part of central metabolism and are not expected to play any role in the induction of the rhamnose pathway itself. Lactaldehyde dehydrogenase is involved in the conversion of L-lactaldehyde to L-lactate. Several putative lactaldehyde dehydrogenases (AldA) based on a characterized AldA in *E.coli* were found in *A. niger* [31]. The first putative gene (NRRL3_11302) shares 33.3% identity with AldA from *E. coli* and it is significantly up-regulated in the reference strain compared to all the deletion mutants. Also, one putative lactate dehydrogenase (LDH; NRRL3_07593) that catalyzes the reaction from lactate to pyruvate, shares 37.4% identity with a characterized LDH from *Rhizopus oryzae* [32]. RNA-seq results showed that this putative gene is not induced after 2 h of transfer. This potentially leaves us with two options for the identity of the inducer, viz. L-KDR or 2, 4-diketo-3-deoxy-L-rhamnonate, and further work will be required to discriminate between these two options.

## Conclusion

In conclusion, this study confirmed the function of *lraA*, *lraB* and *lraC* in the *A. niger* L-rhamnose catabolic pathway in vivo. Our search for candidate genes for the L-KDR aldolase (*lraD*) resulted in selection and deletion of three genes, but none of them showed to have a role in the L-rhamnose pathway. Based on further conserved domain searches with previously characterized L-rhamnose metabolic genes, we suggest that L-KDR may be converted via an unknown aldolase or via an unknown KDRDH.

Gene expression profiling in the wild-type and the L-rhamnose metabolic mutants demonstrated that L-rhamnose, L-rhamnono-γ-lactone and L-rhamnonate are not the inducers of the RhaR-regulated genes. The very low expression levels of the first two metabolic genes (*lraA* and *lraB*), three RGI-specific genes (*rglB*, *rgxA*, *rgae*A) and *rhaR* observed in Δ*lraC* in the presence of L-rhamnonate showed that the third step is necessary to generate an inducer in order to activate these genes. In summary, these results thus strongly suggest that the L-rhamnose pathway intermediate L-KDR is the real inducer of RhaR-regulated genes in *A. niger.*

## Additional files

Additional file 1: Table S1. Strains used in this study. (PDF 104 kb)

Additional file 2: Table S2. Primers used in this study. Overlapping sequences for fusion PCR are written in bold. (PDF 80 kb)

Additional file 3: Table S3. Primers used in this study to generate the gene fragments for qRT-PCR analysis. (PDF 252 kb)

Additional file 4: Table S4. Identification of candidate LraD genes in *Aspergillus niger.* The data are organized according to the fold changes (highest to smallest). The fold change is the difference in expression between the wild-type on rhamnose over glucose. *p*-value <0.05 (cells marked yellow). In red are indicated the three LraD candidates selected. (XLSX 17 kb)

Additional file 5: Table S5. Putative transporter genes in *A. niger* and their expression levels in the Δ*lraA*, Δ*lraB*, Δ*lraC*, Δ*rhaR* mutants and the reference strain after a 2 h transfer to L-rhamnose. The expression levels are mean values of duplicate samples. Genes with values higher than 120 FPKM are considered highly expressed and marked red. Genes with values lower than 20 are considered low expressed and marked green. The fold change is the difference in expression between the reference strain and the deletion mutants. The cut-off for differential expression is fold change >1.5 (cells marked red if up-regulated and green if down-regulated) and p-value <0.05 (cells marked yellow). (XLSX 524 kb)

Additional file 6: Table S6. Genes of *A. niger* encoding pectinolytic enzymes and their expression in Δ*lraA*, Δ*lraB*, Δ*lraC*, Δ*rhaR* mutants and the reference strain after a 2 h transfer to L-rhamnose. The expression levels are mean values of duplicate samples. Genes with values higher than 120 are considered highly expressed and marked red. Genes with values lower than 20 are considered low expressed and marked green. The fold change is the difference in expression between the reference strain and the deletion mutants. The cutoff for differential expression is fold change >1.5 (cells marked red if upregulated and green if downregulated) and p-value <0.05 (cells marked yellow). HGA = homogalacturonic acid, XGA = xylogalacturonan, RG-I = Rhamnogalacturonan-I, SC = side chains. (XLSX 624 kb)

## Abbreviations

CM: complete medium
GEO: Gene Expression Omnibus
HGA: Homogalacturonan
L-KDR: 2-keto-3-deoxy-L-rhamnonate
MM: Minimal medium
RG-I: Rhamnogalacturonan I
RG-II: Rhamnogalacturonan II
RhaR: L-rhamnose responsive transcription factor
RhtA: L-rhamnose transporter
XGA: Xylogalacturonan
