# Supplementary material for: In vivo functional analysis of L-rhamnose metabolic pathway in Aspergillus niger: a tool to identify the potential inducer of RhaR
Source: BMC Microbiol. 2017 Nov 6;17:214. doi: 10.1186/s12866-017-1118-z (PMC5674754; doi:10.1186/s12866-017-1118-z)
Supplement: Supplementary file 1 — Strains used in this study. (PDF 104 kb) [file 12866_2017_1118_MOESM1_ESM.pdf]

Table S1. Strains used in this study

**Strain**	**CBS accession number**	**Genotype**	**Description**	**Reference**
FP-1132.1 (reference)	CBS 141257	*cspA1, pyrG$^-$::AOpyrG, kusA::amdS*	Restored pyrG in N539.20	(Alazi *et al.*, 2016)
Δ*lraA*	CBS 141252	*cspA1, pyrG$^-$, kusA::amdS, lraA::AOpyrG*	Δ*lraA* in N539.20	This study
Δ*lraB*	CBS 141253	*cspA1, pyrG$^-$, kusA::amdS, lraB::AOpyrG*	Δ*lraB* in N539.20	This study
Δ*lraC*	CBS 141254	*cspA1, pyrG$^-$, kusA::amdS, lraC::AOpyrG*	Δ*lraC* in N539.20	This study
Δ*rhaR*	CBS 137440	*cspA, ΔkusA::amdS$^+$,pyrG$^-$,ΔrhaR ::pyrG$^+$*	Δ*rhaR* in N539.20	(Gruben *et al.*, 2014a)
