# Supplementary material for: In vivo functional analysis of L-rhamnose metabolic pathway in Aspergillus niger: a tool to identify the potential inducer of RhaR
Source: BMC Microbiol. 2017 Nov 6;17:214. doi: 10.1186/s12866-017-1118-z (PMC5674754; doi:10.1186/s12866-017-1118-z)
Supplement: Supplementary file 2 — Primers used in this study. Overlapping sequences for fusion PCR are written in bold. (PDF 80 kb) [file 12866_2017_1118_MOESM2_ESM.pdf]

Table S2. Primers used in this study. Overlapping sequences for fusion PCR are written in bold.

Primer name	Sequence (5' to 3')	Used for
pyrG up f	gtaacgccagggttttcccagtcacgacgGATTCCTCGAGCTAACATACATTC	amplification of *pyrG* 5'flank
pyrG up r	**agcacttaccttcgcattttctggtatatt**ATGGAAAGAGGATTGTATAATGAGAG	amplification of *pyrG* 5'flank
pyrG down f	**cctgtgttggttctcaggaactgcgaatat**GATGGTTATGAATGATATAGAAATGC	amplification of *pyrG* 3'flank
pyrG down r	gcggataacaatttcacacaggaaacagcCGAGTTTCTTTTATCTAACTTGTCG	amplification of *pyrG* 3'flank
lraA up f	gtaacgccagggttttcccagtcacgacgTGAGCTGGTCAAGCGTTGTGAG	amplification of *lraA* 5'flank
lraA up r	**ccttcgcattttctggtatattTCCAAC**GATTGTGGTTGATGGTCGATTGG	amplification of *lraA* 5'flank
lraA down f	**ggttctcaggaactgcgaatatTCCAAC**GGGCGCAGATACTTGTGGATGG	amplification of *lraA* 3'flank
lraA down r	ggttctcaggaactgcgaatatTCCAACGGGCGCAGATACTTGTGGATGG	amplification of *lraA* 3'flank
lraB up f	gtaacgccagggttttcccagtcacgacgTTTAGGCCATCGCTCTCGAATAC	amplification of *lraB* 5'flank
lraB up r	**ccttcgcattttctggtatattTCCAAC**GTACACCGATCGCCATAACCAAG	amplification of *lraB* 5'flank
lraB down f	**ggttctcaggaactgcgaatatTCCAAC**TTTCTTTGTTTGTGTTCGTGTTTCG	amplification of *lraB* 3'flank
lraB down r	gcggataacaatttcacacaggaaacagcGGCATTATCAATAGGCACAGTCATC	amplification of *lraB* 3'flank
lraC up f	gtaacgccagggttttcccagtcacgacgCGCATTTGACCTTCCTTTGCTTAC	amplification of *lraC* 5'flank
lraC up r	**ccttcgcattttctggtatattTCCAAC**GTTGGCAGTAGTTTAGCGGAGTGG	amplification of *lraC* 5'flank
lraC down f	**ggttctcaggaactgcgaatatTCCAAC**GCAATGGAACAATCCTCGACATG	amplification of *lraC* 3'flank

lraC down r	gcggataacaatttcacacaggaaacagcCTGATGCCGTATTTGCCCAATG	amplification of *lraC* 3’flank
lraA gene f	GCGAGCAGACACAAGCAAATG	To check the presence/absence of the *lraA* gene
lraA gene r	CCGCACACGACTGCATAAGAC	
lraB gene f	ATCCACCTCTTCCCAGCATCC	To check the presence/absence of the *lraB* gene
lraB gene r	GTACGGCAGACGAAGGTTTGG	
lraC gene f	TGGCTATCTGGGACCTTCTGG	To check the presence/absence of the *lraC* gene
lraC gene r	TGGAAGGGTGTATTGGGTTGC	
