# Supplementary material for: In vivo functional analysis of L-rhamnose metabolic pathway in Aspergillus niger: a tool to identify the potential inducer of RhaR
Source: BMC Microbiol. 2017 Nov 6;17:214. doi: 10.1186/s12866-017-1118-z (PMC5674754; doi:10.1186/s12866-017-1118-z)
Supplement: Supplementary file 3 — Primers used in this study to generate the gene fragments for qRT-PCR analysis. (PDF 252 kb) [file 12866_2017_1118_MOESM3_ESM.pdf]

Table S3. Primers used in this study to generate the gene fragments for qRT-PCR analysis

**Gene**	**Gene number**	**Forward**	**Reverse**
Histone (*H2B*)	NRRL3_09214	AGACCTCTGTGAGGCTCATCCT	AAGCACGCGGTGTCGG
lraA	NRRL3_01494	AAGCCGTGGAAGCCTTTGGAC	GTGAGTAATGCGGGAGTGTGC
lraB	NRRL3_01493	GCAGCCCGAGTTCATTGATGG	GCAGTTGGCGTTTGATCTGGG
lraC	NRRL3_01495	TCCTGGGCTCTTTCTGCGTC	GACTAAGGGATTTGCCACGGG
lra4	NRRL3_ 08779	CTCGCCCAGTCCAACTCCAAT	AGCTCACTTGCGGAAATGTTGG
rhaR	NRRL3_01496	TCCACGGGTCCACAATAGCC	CCCCGACATCCTCTTCTTCGT
rgaeA	NRRL3_00169	ACGTCGCCCAATACCTGAGC	CTACGGCTTGGTTGGACACG
rgxA	NRRL3_02832	CCACTTCAGAAACATCAGCGGC	GGGTAATCAGTCACCGTTGCG
rglB	NRRL3_10115	GGAAAAGGCTCCACTGCGTCT	CAGCTCGGATCTGCCAGTGTT
